# A Novel, Low-Volume Method for Organ Culture of Embryonic Kidneys That Allows Development of Cortico-Medullary Anatomical Organization

**DOI:** 10.1371/journal.pone.0010550

**Published:** 2010-05-10

**Authors:** David D. R. Sebinger, Mathieu Unbekandt, Veronika V. Ganeva, Andreas Ofenbauer, Carsten Werner, Jamie A. Davies

**Affiliations:** 1 Max Bergmann Center of Biomaterials, Leibniz-Institut für Polymerforschung Dresden, Dresden, Germany; 2 Centre for Integrative Physiology, University of Edinburgh, Edinburgh, Scotland; Katholieke Universiteit Leuven, Belgium

## Abstract

Here, we present a novel method for culturing kidneys in low volumes of medium that offers more organotypic development compared to conventional methods. Organ culture is a powerful technique for studying renal development. It recapitulates many aspects of early development very well, but the established techniques have some disadvantages: in particular, they require relatively large volumes (1–3 mls) of culture medium, which can make high-throughput screens expensive, they require porous (filter) substrates which are difficult to modify chemically, and the organs produced do not achieve good cortico-medullary zonation. Here, we present a technique of growing kidney rudiments in very low volumes of medium–around 85 microliters–using silicone chambers. In this system, kidneys grow directly on glass, grow larger than in conventional culture and develop a clear anatomical cortico-medullary zonation with extended loops of Henle.

## Introduction

This paper describes a method for organ culture of developing kidneys that improves on conventional methods in terms of both economy and organotypic realism.

Organ culture of embryonic kidney rudiments has been established for almost ninety years. The earliest methods suspended renal rudiments in Carrell flasks in semi-solid media, such as clotted owl plasma [1]–[6]. The clot system, which yielded good though variable development, was replaced in the 1960s by a simpler system in which kidney rudiments are grown on filters supported on a metal grid at a gas-medium interface [7]–[9]. This is the system generally used to this day, with the occasional variation of using filter inserts designed for multiwell plates, still at a gas-medium interface, in place of pieces of filter supported on steel grids. The filter supports are used because culture in simple liquid hanging drops, which works well for other organs such as salivary glands, does not work well for kidneys and their development in such systems is very poor.

Organ culture of kidney rudiments has been, and continues to be, very valuable in the study of renal development [3]; [10]–[13]. The system has been used to study the dynamics of normal development by time-lapse photography, initially by brightfield microscopy and more recently with fluorescent reporter proteins [8]; [14]. It has been used to study the developmental functions of specific molecules by experimental addition of exogenous growth factors [15], function-blocking antibodies [16], vitamins [17], oligosaccharides [18], drugs [19], antisense oligonucleotides [20] and short interfering RNAs [21]; [22]. It has also been used to test the cell autonomy of mutations by production of chimaeric recombinant kidneys [23].

Useful as it is, the established culture system suffers from a number of limitations. It requires volumes of media of the order of millilitres, which limits its use in high-throughput screens that require high concentrations of expensive reagents such as siRNAs, and it requires supporting filters that are significantly harder to modify with custom substrates than is glass. Also, while cultured kidneys show good development of the branched collecting duct system and of nephrons to the S-shaped stage and beyond, including differentiation of specific regions such as proximal tubule, distal tubule etc, they do not show development of a distinct renal medulla into which Loops of Henle extend. In conventional culture, the loops of Henle do not form [24] while in culture systems that optimize the maturation of nephrons, such as those using hyaluronic acid, loops of Henle form but are arranged haphazardly rather than extending into the medulla [25].

In this paper, we describe a simple culture system that allows kidney rudiments to be cultured directly on glass coverslips in just 85 µl of medium. The development of these kidneys is superior to traditional methods when compared by any of the usual metrics (overall size, nephron number and the extent of ureteric bud branching) and they show correct cortico-medullary zonation. This new technique therefore offers considerable advantages, of economy and realism of development, over the established method.

## Materials and Methods

### Organ culture

Organ rudiments were microdissected from E11.5 NMRI or CD1 mouse embryos; they were pooled and assigned randomly to control or experimental groups. For conventional culture, the rudiments were placed on 5 µm pore-size polycarbonate filters at the bottom of a well insert in a six well plate (Corning, Costar), or on top of a stainless steel Trowell grid in a 3.5 cm culture dish in kidney culture medium (KCM: Eagle's minimal essential medium with Earle's salts and non-essential amino acids (GIBCO), 10% foetal bovine serum (Biochrom/Biosera) and 1% penicillin/streptomycin (Sigma)). For some experiments, 1 nM TGF-β (Sigma T7039) or 100 ng/ml GDNF (Sigma G1777) were added.

Low-volume cultures used sterilized silicone rings (flexiPERM Cone shape A, Greiner BioOne), on 22×22 mm coverslips (Menzel Gläser, Germany), cleaned in 5∶1∶1 H_2_O∶H_2_O_2_∶NH_4_OH (10 min, 70°C), in tissue culture dishes (35×10 mm, Sarstedt/Greiner). Kidney rudiments were placed close to the middle of the circle; medium carried over in this pipetting operation was removed and replaced by the final KCM culture medium (70–200 µl), the complete enclosed area of the coverslip being wetted. The dish surrounding the silicone ring was filled with PBS containing 1% penicillin/streptomycin). All cultures were incubated at 5% CO2 at 37°C, medium being changed every 2 days.

For time lapse movies, cultures were placed in the incubation chamber of a Zeiss Axiovert 200 microscope and images were taken every 10 or 15 min. NIH ImageJ 1.37v. was used to create time lapse movies. Phase contrast images of living cultures were taken every 12 h using an Olympus IX50 microscope.

### Fixation and immuno/lectin fluorescence

Kidney rudiments were fixed in methanol at −20°C, washed in PBS 10 min, then incubated in primary antibody in PBS, overnight at 4°C. Primary antibodies were anti-laminin (1∶100 Sigma L9393), anti-calbindin D28k (1∶100 Sigma C9848),), anti-megalin (1∶150, obtained from Thomas Wilnow), anti-human-Wilms' Tumour 1 (1∶50, Dako M3561), anti-Pax-2 (1∶100, Covance PRB-276P) and anti-E-cadherin (1∶100, BD Transduction Laboratories 610181). Samples were washed for at least 30 min in PBS then incubated with appropriate secondary antibodies overnight at 4°C. The secondary antibodies used were: anti-rabbit IgG - TRITC (1∶100, Sigma T6778), anti-mouse IgG–TRITC (1∶100, Sigma T5393) and anti-mouse IgG - FITC (1∶100, Sigma F6257). For antibody/lectin co-stainings, all solutions contained 1% milk powder in PBS and 10 ng/ml lectin from *Dolichos biflorus*-FITC (Sigma L9142) was included with the secondary antibody. Finally the samples were washed in PBS and mounted on slides.

### Cell proliferation and apoptosis detection assay

BrdU (5-bromo-2-deoxy-uridine) was added to the medium of kidney rudiments in culture 4 hours before fixation to a final concentration of 100 µM. Detection was performed as described by [26] except that cell death detection master mix (In Situ Cell Death Detection Kit, TMR red, Roche 12156792910) was added along with primary antibodies. Samples were washed for 30 min in PBS and incubated in secondary antibodies overnight at 4°C. After a PBS wash, 1 µg/ml DAPI (Sigma) in PBS was added for 20 min. Finally the samples were washed in PBS and mounted on slides.

### Morphometric quantification

Immunostained samples were examined on a confocal laser scanning microscope (TCS SP5, Leica Microsystems, Wetzlar, Germany). Serial 5-µm optical sections of each kidney were acquired. FITC, TRITC and DAPI emissions were acquired sequentially. Ureteric bud tips and nephrons were counted manually. Area was defined manually and measured using NIH ImageJ 1.37v (http://rsb.info.nih.gov/nih-image/).

### Measurement of cytokines

Right and left kidneys of the same embryo were dissected and allocated randomly to either conventional culture or the new method. Supernatants conditioned from 0–2 d 2–4 d were collected and diluted in fresh medium to a standard total of 3 ml. Expression of 40 cytokines was assessed using R&D systems' mouse cytokine array panel according to the manufacturer's directions. Experiments were performed in triplicate.

### Surface tension measurements

DOPC (1,2-dioleoyl-sn-glycero-3-phosphocholine; Sigma P6354) was added to and dispersed at 1 mg/ml in KCM by 30 min. of sonication on ice. Surface tension was determined using the pendant drop-method based on the Young-Laplace equation [27]. After calibrating the system (Contact Angle System OCA 30, DataPhysics Instruments, Germany) with degassed water, the surface tensions of KCM and KCM with DOPC were measured.

### Animals

The animals from which tissue samples were obtained, bred and kept according to relevant UK Home Office guidance (http://scienceandresearch.homeoffice.gov.uk/animal-research/legislation/index.html) and the Animals (Scientific Procedures) Act, 1986, available from the same website: they were killed by trained technical staff according to a method listed in Schedule 1 of that Act. Ethical approval for keeping the animals and obtaining these tissue samples was approved by the University of Edinburgh's local ethics approvals process.

## Results

### Kidney rudiments develop well on glass if cultured in low volumes of medium

**Figure 1:**
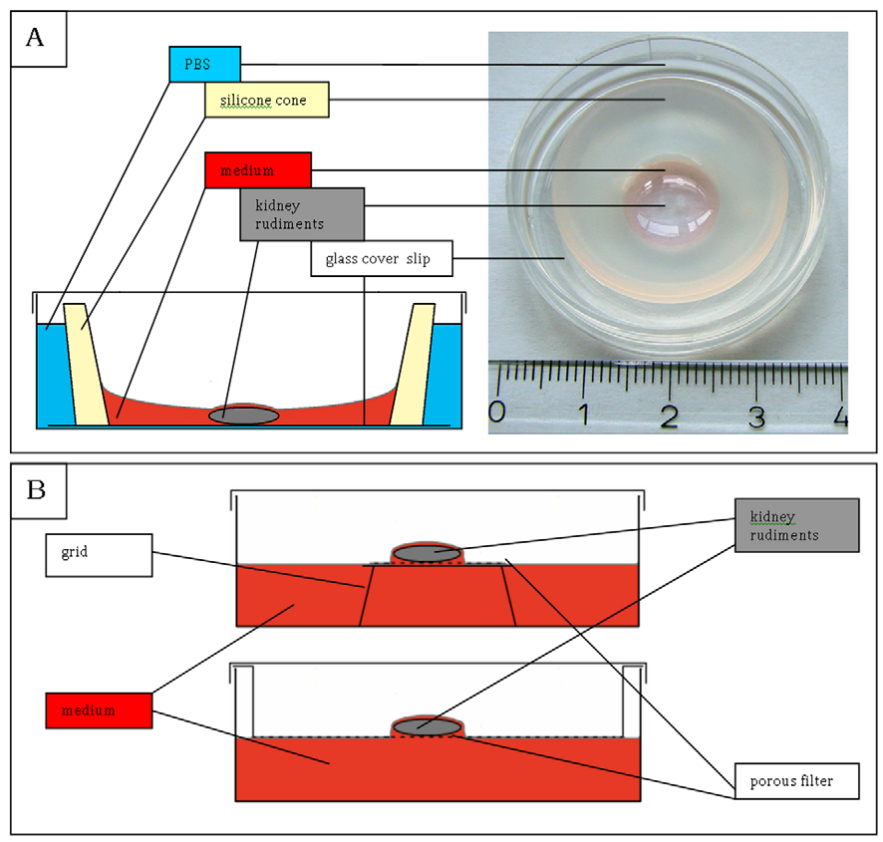
Methods for embryonic kidney culture. (A) The low-volume culture method described in this paper, drawn from the side and photographed from above. (B) Conventional, high volume culture either on a membrane on a Trowell grid (above) or on the membrane at the bottom of a well insert (below). The blue in (A) depicts PBS, the red in all diagrams depicts culture medium, yellow in (A) symbolizes the silicone ring and dark grey the embryonic kidney rudiments. The numbered divisions on the ruler are centimetres.

We began this work with the aim of growing embryonic kidney rudiments on glass coverslips that could be coated easily with defined matrix components. To define a small culture area on the glass, we used FlexiPERM silicone rings, each with the approximate shape of a decapitated cone, the smallest end of which defined a 1 cm^2^ (i.e. 5.6 mm radius) circle (Fig 1A). It was at once clear that kidney rudiments did not grow well when supplied with large volumes of media in this system. Smaller volumes, of 85, 90, 120 or 200 µl were therefore tried (volumes of 70 µl and less were found to be insufficient to wet the whole circle permanently). Development of the organ rudiments after four days in each condition was quantified by measuring rudiment area, number of ureteric bud tips (2 at the time of isolation) and number of nephrons (0 at the time of isolation).

**Figure 2:**
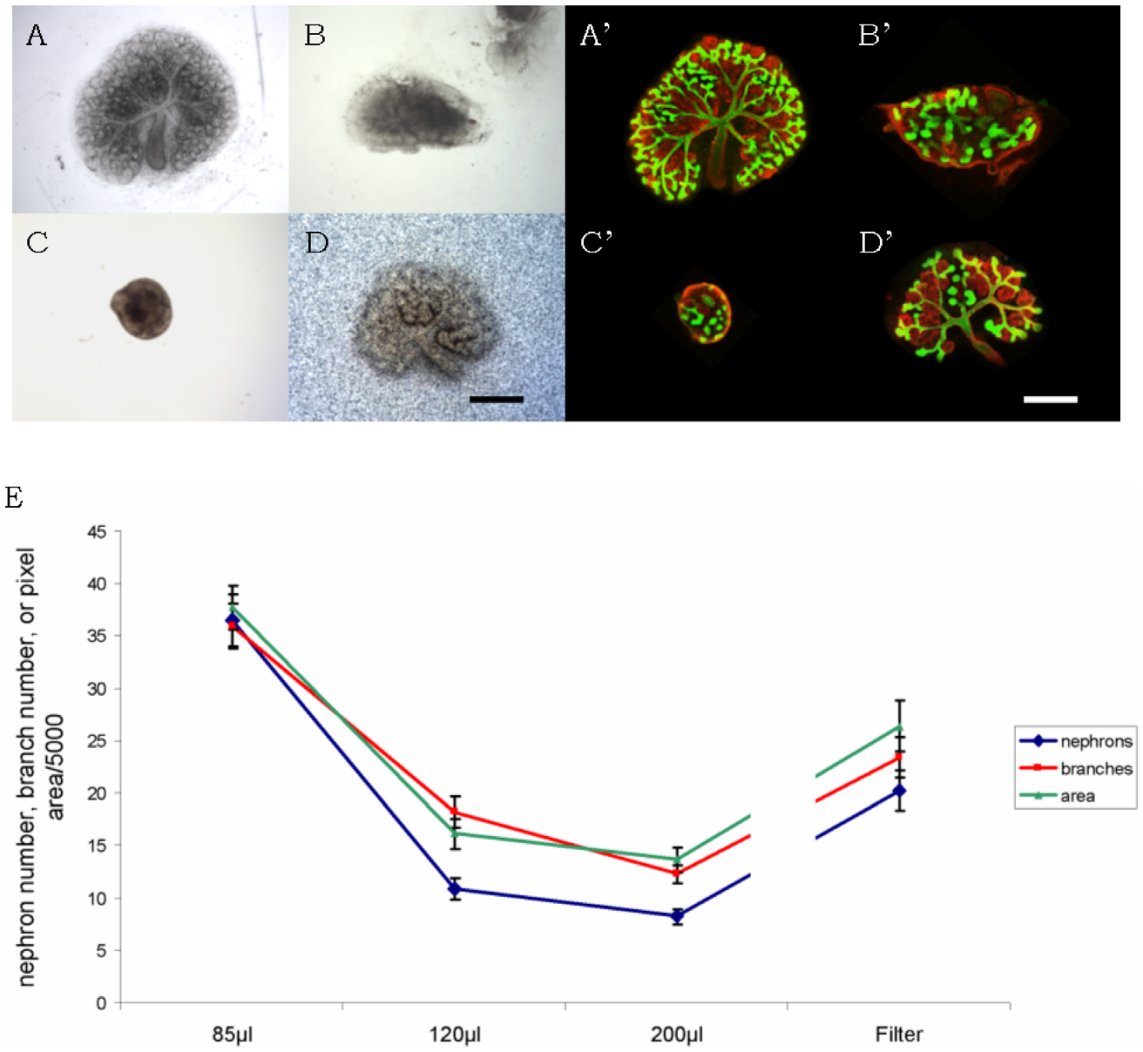
Development of mouse kidney rudiments in conventional culture and on glass inside silicone rings. (A–C) show phase contrast views of kidney rudiments grown for 4 days in silicone rings on glass in 85 µl, 120 µl and 200 µl respectively, while (D) shows a kidney grown in the conventional system (on a filter on a Trowell grid: the ‘noise’ in the background is the filter). On glass, the lowest volume, 85 µl, shows the best development, resulting in a larger kidney than the conventional system. (A′–D′) show kidneys grown in the same conditions as (A–D) but stained for basement membrane marker laminin (red) and the ureteric bud marker calbindin-D_28k_ (green). (E) shows a quantitative analysis of area, nephron and bud tip numbers for each of these culture conditions. Error bars depict standard errors of the mean and are derived from at least 49 kidneys in total, from six different runs of the experiment, each run using between 6 and 18 kidneys. Scale bars  = 500 µm.

Control kidneys, cultured by the conventional Trowell method (Fig 1B) developed normally, showing a calbindin-positive ureteric bud that was well-branched (mean 23.4, σ = 16, n = 49) and many developing nephrons (mean 20.2, σ = 16, n = 49), identifiable by their shapes and by their laminin-rich basement membranes and absence of calbindin staining (Fig 2D, D′). In 200 µl medium, many times deeper than the height of the kidney, kidneys remained rounded and developed poorly (Fig 2C, C′), showing less than half the ureteric bud branches and nephrons of control kidneys grown on conventional Trowell filters. This was expected from previous reports (see Introduction). With lower volumes of medium, however (Fig 2A, A′, B, B′), the extent of development improved (Fig 2E). In the optimum volume - 85 µl - kidney rudiments covered significantly more area (42%) than did filter-grown controls and they produced 46% more ureteric bud tips and 81% more nephrons. The variability between kidneys was also reduced (standard deviations in measurements of area, branch number and nephron number were all only about four fifths as large, as a proportion of the means to which they applied, as they were for filter-grown controls). Adding 500 µl of culture medium to kidney rudiments that had already been cultured in 85 µl for 1 day of culture caused the kidneys to round up and to cease developing well: they therefore require low volumes continuously, and not just to promote initial settling on the glass.

**Figure 3:**
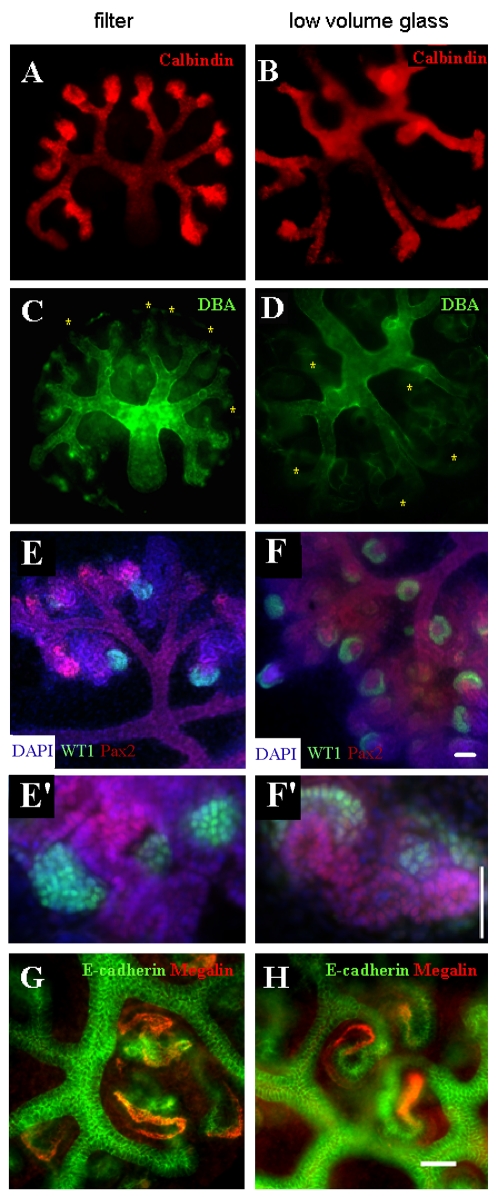
Normal segmentation of nephrons grown on filters (A, C, E, G) and in low volumes on glass (B, D, F, H). (A, B) show kidneys stained for calbindin, which stains the whole ureteric bud; (C, D) show kidneys stained for Dolichos biflorus agglutinin (DBA), which stains only the bud stalk–the ‘missing’ tips visible in A,B but not in C,D are marked with yellow stars; (E, F) show kidneys stained for the WT1, which is expressed strongly in crescents that consist of developing podocytes: in these images, staining for Pax2, expressed in bud, condensates and early nephrons, is used to reveal the general structure of the rudiment and to place the developing podocytes in their anatomical context: higher power views are shown in E′ and F′, revealing the nuclear location of WT1. (G, H) show higher power views of kidneys stained for the ureteric bud and distal tubule marker, E-cadherin, and the proximal tubule marker megalin; the expression of each of these markers is similar in both culture systems. Scale bars  = 50 µm; the scale bar shown in F applies to A–F, that shown in F′ to E′ and F′, and that shown in H applies to G and H.

**Figure 4:**
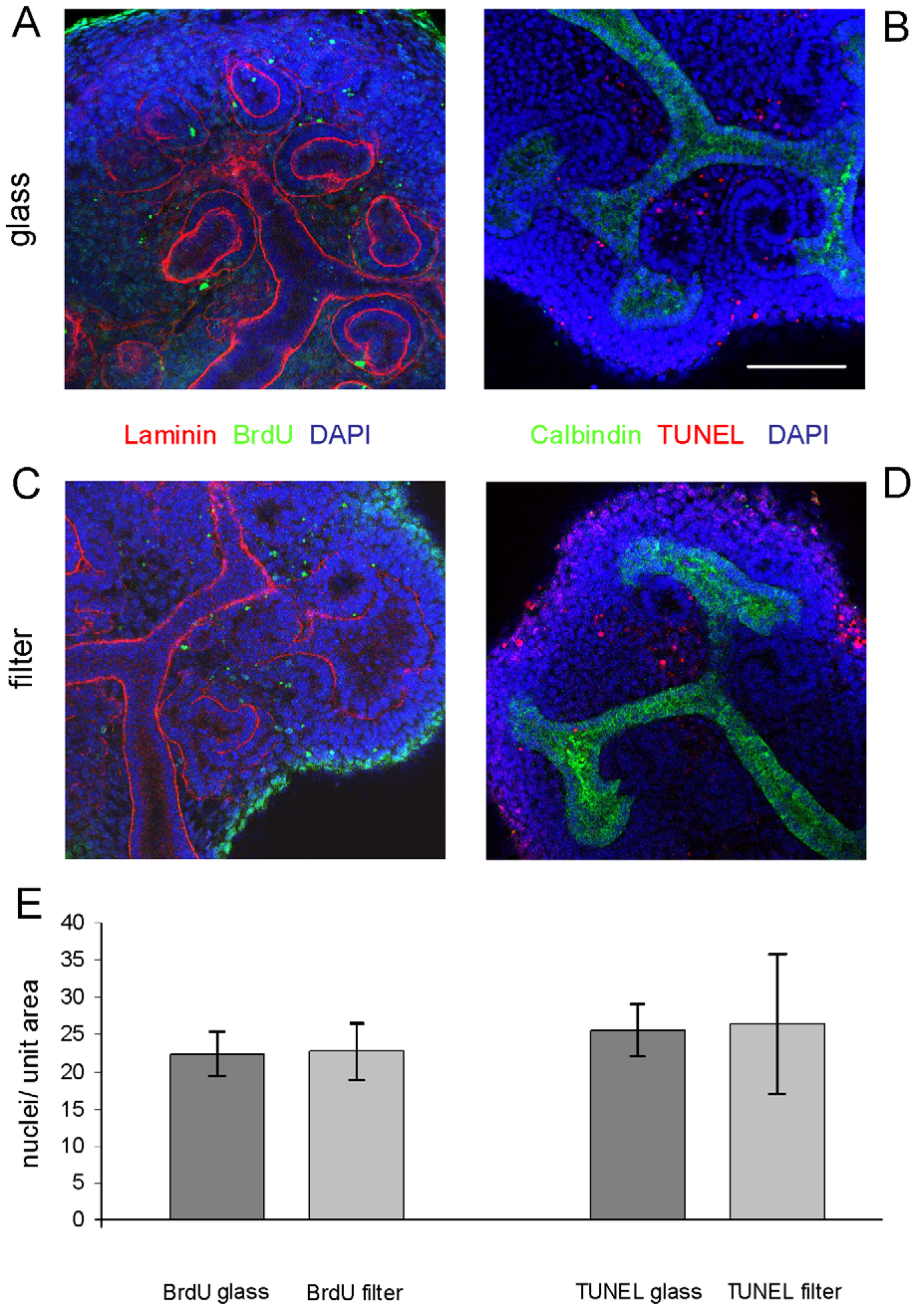
There is no significant difference after two days in rates of proliferation or cell death in nephrons formed in the two culture systems. Proliferation was measured using BrdU incorporation and apoptosis by terminal end labelling (TUNEL); source images typical of those analysed quantitatively are shown for BrdU (A glass; C filter) and TUNEL (B glass; D filter). Over many such images, the numbers of BrdU or TUNEL-positive nuclei per unit area of visible nephron cross-section were counted. The data, from ten filter-grown kidneys and thirty one glass-grown ones, (E) show no evidence for difference between culture systems (a two-tailed Student's t-test yields p = 0.93 and p = 0.94 for BrdU and TUNEL respectively). Scale bar is 100 µm.

The nephrons and ureteric bud branches of kidneys grown in conventional organ culture showed patterns of gene expression similar to those observed *in vivo*, as has been described before [28]–[31]. The ureteric bud, for example, expressed calbindin D_28K_ (Fig 3A) and was divided into a stalk region that bound *Dolichos biflorus* lectin and a tip region that did not (Fig 3C) [32]; [33] (Fig 3C). The nephrons showed correctly-restricted expression of markers; for example, the most proximal ends of the nephrons differentiated into podocytes that expressed high levels of WT1 (Fig 3E: the weak expression of WT1 in condensing mesenchyme is normal and reflects an earlier role for that protein in nephron development [21]), the proximal tubules expressed megalin and the distal tubules and ureteric bud expressed E-cadherin (Fig 3G). Nephrons and ureteric buds of kidneys grown using our low volume culture system showed the same organotypic expression of segment marker genes. Ureteric buds were divided correctly into tip and stalk zones (Fig 3B, D), developing podocytes expressed WT1 (Fig 3F), proximal tubules expressed megalin and distal tubule expressed E-cadherin (Fig 3H). Rates of cell proliferation and apoptosis in nephrons, measured by BrdU incorporation and Terminal deoxynucleotidyl transferase dUTP nick end labelling (TUNEL) respectively, were not significantly different in the two culture systems after two days (Fig 4).

### Kidney rudiments grown in the low volume system show typical responses to positive and negative morphogens

Conventional kidney organ culture has been used to identify various diffusible regulators of renal development, by applying suspected regulators to the medium and observing any changes to subsequent morphogenesis. We have tested kidneys growing in the low volume system for their response to two known modulators of morphogenesis, one positive and one negative. This had two purposes; (a) to verify the normality of development and (b) to check that the culture volume used is not so small that these regulators become quickly exhausted or inactivated by cellular secretions. The factors we used were TGF-β, a known inhibitor of ureteric bud branching [34] and GDNF, a known activator [35].

**Figure 5:**
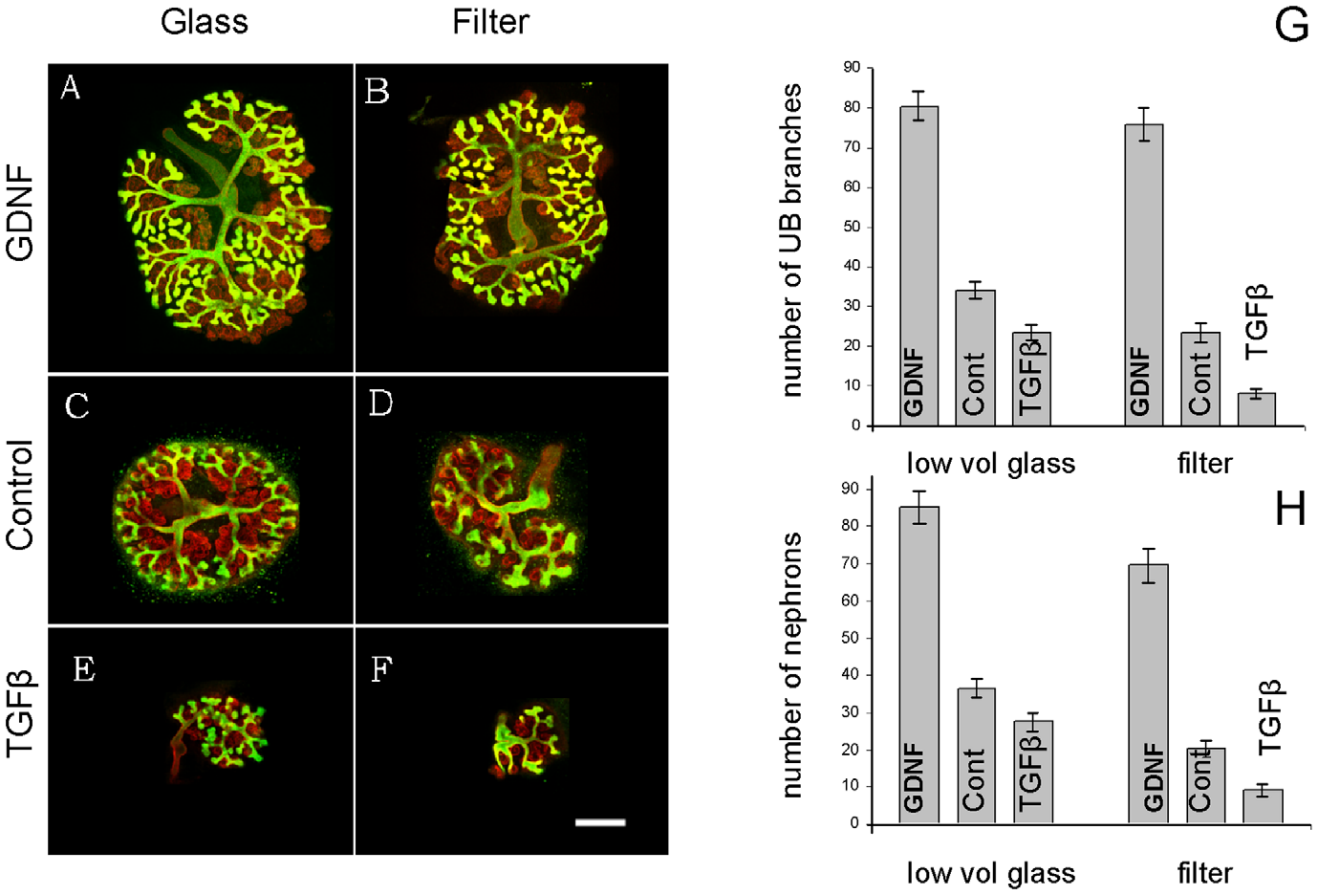
Kidneys grown in the low volume glass system show normal responses to known morphogens. (A, B) show the enhanced development of kidneys grown in 100 ng/ml GDNF in the low volume glass system and on conventional filters, stained for laminin (red) and calbindin-D_29k_ (green). (C, D) show kidneys grown in control medium and (E, F) show the decreased development of kidneys cultured in the presence of 1 nM TGFβ. (G) shows the effects of these molecules on ureteric bud (UB) branching, in the low volume and the filter systems and (H) shows their effects on nephron number. Each bar represents data from between 10 and 71 kidneys. All values in (G) and (H) differ significantly from their respective controls, by a two-tailed Student's t-test assuming unequal variances, the weakest difference (that between nephrons in control and TGFβ, on glass) still having a p-value of 0.009. Scale bar 500 µm.

Exogenous TGF-β, applied at 1 nM for four days [36], had similar effects on the development of kidneys in conventional and low volume culture, although the inhibitory effects were a little less dramatic in the low volume method (Fig 5). Exogenous GDNF, added at a final concentration of 100 ng/ml again for four days [36] significantly increased branching morphogenesis in both systems so that more than a doubling of the amount of nephrons and ureteric bud branches was achieved.

### Kidney rudiments develop cortico-medullary zonation when cultured in this system

Kidney rudiments developing in conventional organ culture show excellent recapitulation of *in vivo* development at a fine scale but one important large-scale aspect of development is typically lost, or at least seriously under-developed. *In vivo*, growth and extension of stalks of the ureteric bud and the Loops of Henle divides the kidney into two broad zones, an outer cortex containing the glomeruli and ureteric bud tips, and an inner medulla that is dominated by straight tubules of the collecting duct system and the loops of Henle. This organization is critical to the urine-concentrating activity of the metanephros, so lack of a good culture model to allow the development of cortico-medullary differences to be studied is a serious limitation to the field.

**Figure 6:**
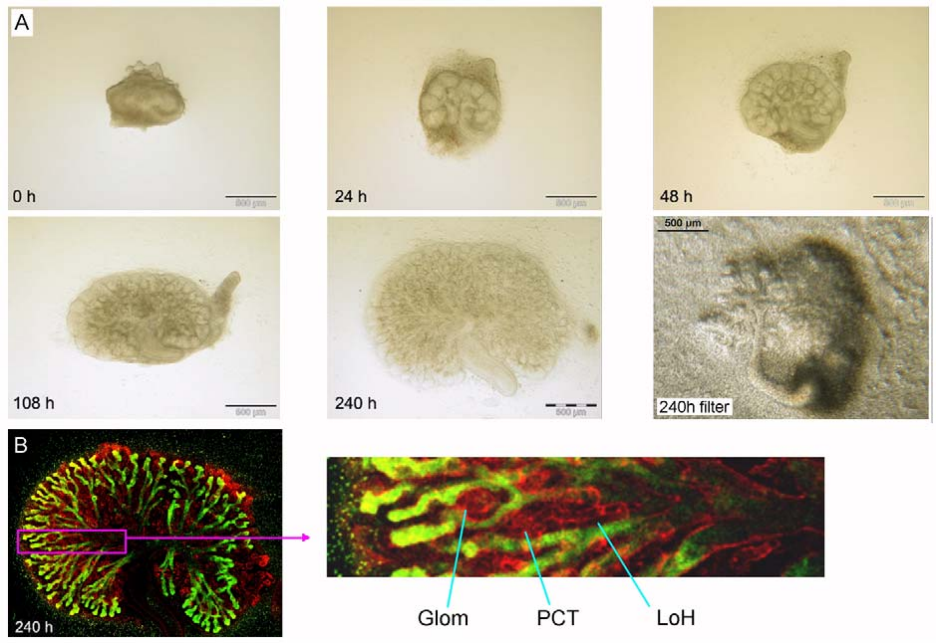
Kidneys grown on glass develop organotypic features including cortico-medially zonation. (A) Shows a time course of development to 240 h (10 d). From about 108 h (4.5 d), the kidney spreads out enough that it begins to divide into two zones, an outer medullary zone that features many nephrons and an inner medulla that contains more elongated tubules (mainly collecting ducts at this stage). By 240 h (10 d), this effect has become more marked. The last panel of (A) shows a kidney rudiment cultured on a filter for 240 h: the organ occupies less area and there is less evidence of corticomedullary zonation. The ‘grain’ in the photograph is an optical effect of the filter pores, which are beneath the kidney; their absence in bright field imaging is another advantage of the glass system. (B) A high-power view of part of a 240 h kidney shows that nephrons (red) are arranged organotypically, with the glomerulus (‘Glom’) and proximal convoluted tubule (‘PCT’) in the cortex and a loop of Henle (‘LoH’) extending down into the medulla, forming a tight hair-slide shape parallel to the collecting ducts (green). Green  =  calbindin, red  =  laminin.

Long-term (10-day) culture in our low volume system resulted in a substantial increase in area of the organ rudiment and the formation of distinct cortical and medullary zones (Fig 6A). The forming glomeruli were restricted to the cortical zone and the medullary zone contained ureteric bud/collecting duct tubules and also loops of Henle (Fig 6B). The new culture system therefore has the substantial advantage, beyond economy with reagents, that it shows more anatomically realistic renal development.

### Kidney rudiments cultured in the low volume system show less evidence for stress than those grown in conventional organ culture

Tissues subject to stress secrete specific cytokines that interact with cells of the immune system to initiate an inflammatory response [37]. These cytokines include tissue inhibitor of metalloproteinase I (TIMP1), monocyte chemotactic protein 1 (MCP1, also called JE), the neutrophil chemokine CXCL1 (also called KC) and interferon gamma (IFNγ) [38]–[41]. Release of these proteins into medium can therefore be used as an indicator for how stressed cells are in culture [42]–[46].

**Figure 7:**
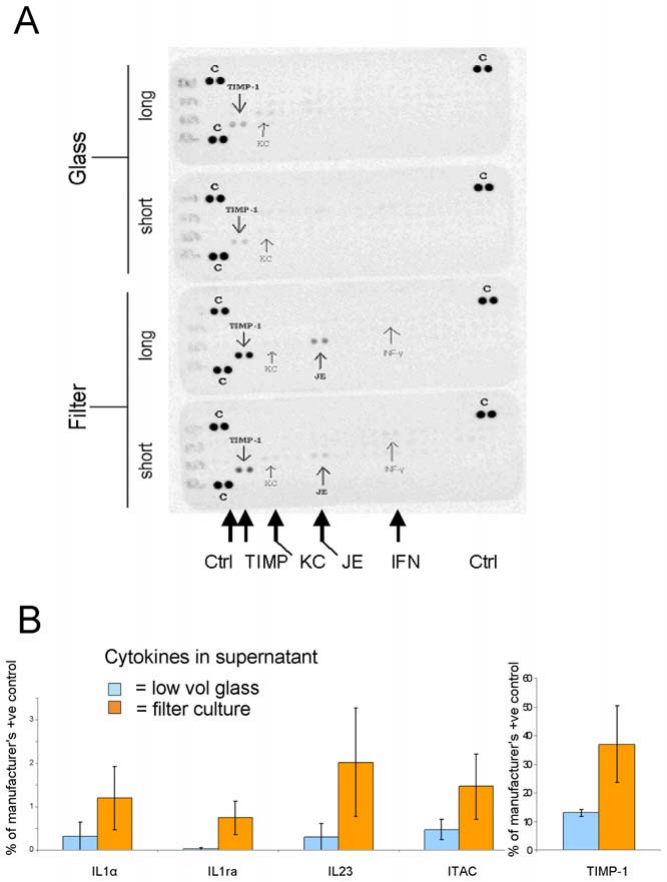
Kidneys grown in the low-volume glass system show less evidence of stress. In (A) the samples labelled ‘short’ are of medium conditioned from day 0–2 and those labelled ‘long’ are from days 2–4. Spots marked ‘C’ are positive controls (to verify the detection kit). The other spots, of TIMP1, KC, JE and IFNγ represent these markers of cellular stress (see main text). (B) shows, quantitatively, the results of these molecules on the arrays, as means of three runs. The Y axis is % relative to the signal provided by the manufacturers' positive control. The right-most part of the graph, showing TIMP (also shown as a spot in A), is plotted to a different y axis. Although the data for each of the molecules, each individual array spot being derived from 3 pooled kidneys and 3 independent arrays being run for each condition, suggests a decrease on glass, the variation in measurement is so large that no one individual molecule has a change that can be regarded as significant at the conventional p≤0.05 (most have p≈0.1). As all molecules are markers for the same physiological state (cellular stress), however, their measurements can be pooled for a one-tailed, paired Student's t-test that uses all 15 experiment-control measurement pairs together (the 15 pairs coming from 5 target molecules x 3 runs each). Viewed this way, the data show that glass culture does produce a significant reduction in production of these stress proteins (p = 0.05). Elements on the spot arrays not shown in this graph showed no significant differences.

Medium from kidneys cultured conventionally in 3 mls of medium contained significant amounts of TIMP1 and MCP1 and smaller but still detectable amounts of CXCL1 (not shown) and IFNγ (Fig 7). Medium from the low volume glass culture system showed lower amounts of TIMP1 and less or equal of the other pro-inflammatory cytokines, measured over either first 48 h or the subsequent 48 h of culture (Fig 7), though the effects were stronger by the second 48 h. These results suggest that the cells in this system were significantly less stressed than those in conventional culture.

### Kidney rudiments may grow better in low culture volumes because of a requirement for effects of surface tension

Something that the conventional high-volume filter method of culture has in common with the low-volume method, but not with the much less effective high-volume methods (including our use of 120 and 200 µl instead of 85 µl the silicone rings), is that the organ rudiment is at the gas-medium interface. There are two reasons that this might be important. One is improved access to oxygen, although this seems unlikely since kidneys grow very well in conventional culture in low (3%) oxygen systems [47]. The other is the effect of the surface itself. Molecules of liquid are mutually attractive. Those in the bulk liquid are surrounded by other molecules on all sides so therefore experience no directional force. Those at a gas-liquid interface surface still have other attractive molecules to the side of them and below them but not above them, so they experience a net average force pulling them back into the bulk liquid. For this reason, surfaces are drawn in until balanced by compressive forces in the bulk liquid, and liquids minimize their free surface area. Actions that would expand the surface area of a liquid work against this tendency to minimize surface are therefore met with an opposing force, usually called surface tension. Where the liquid over a culture substrate is shallow compared to the height of the organ culture itself, the raised profile of the organ forces the surface of the liquid to be larger than it would otherwise be and the organ will experience a flattening force due to surface tension.

**Figure 8:**
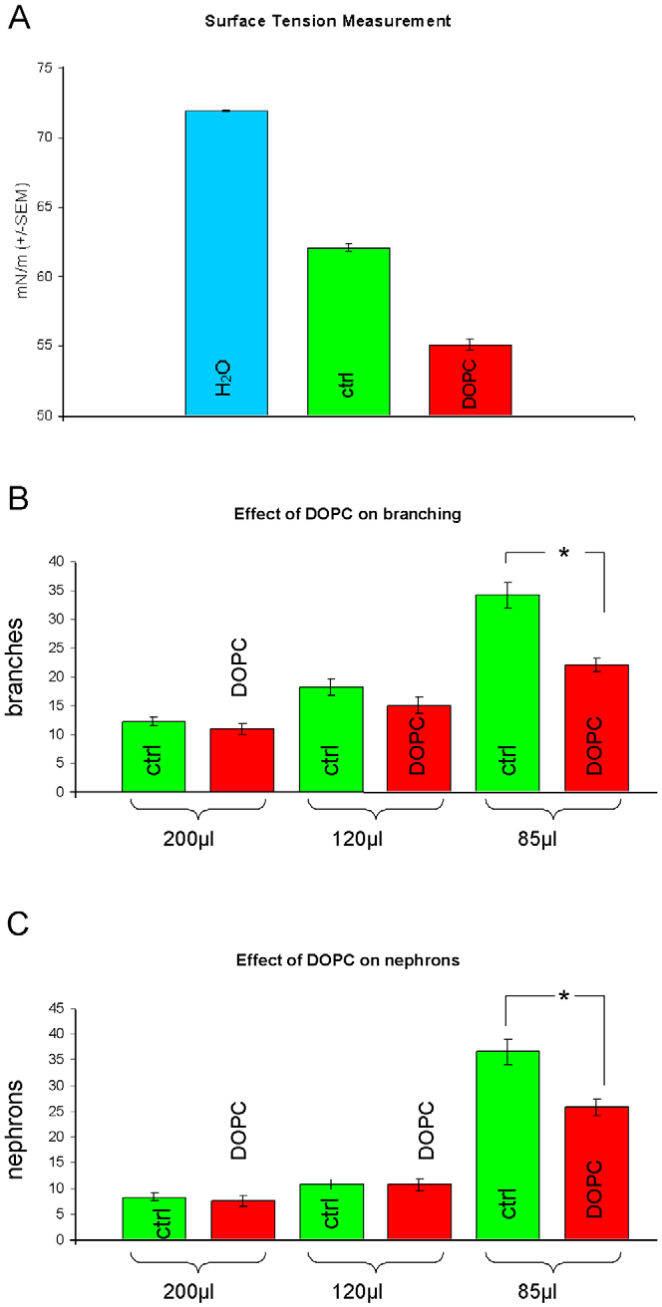
Reduction of surface tension influences development of cultured kidney rudiments. In (A) the reduction of surface tension by the addition of the surfactant DOPC to the medium is demonstrated; data represent means of a minimum of 18 measurements. (B and C) show the influence of the surfactant on ureteric bud branching and amount of nephrons, respectively, if compared to the control; each bar represents data from a minimum of 17 kidneys. The asterisk indicates significant difference (p = 3.4×10^−6^ for branches and 3.3×10^−4^ for nephrons) by a two-tailed Student's t-test assuming unequal variances.

It is therefore possible that the flattening effect of surface tension is important to renal development *in vitro*. We tested this idea by using a surfactant to reduce surface tension in the medium to see if this mimicked the effect of a high volume of medium. The surfactant used, 1,2-dioleoyl-sn-glycero-3-phosphocholine (DOPC), reduces surface tension by forming phospholipid bilayers (rather than the monolayers formed by typical lab detergents) at the air-medium interface [48]. Direct measurements of surface tension in untreated and DOPC treated medium confirmed the surface tension-lowering effects of DOPC (Fig 8A).

The presence of DOPC had no significant effect on the (already poor) development of kidneys under large volumes of medium, as would be expected since these should be relatively free from surface effects (Fig 8B, C). In the low-volume (85 µl) system, though, the presence of DOPC significantly reduced the renal area, the number of ureteric bud tips formed and the number of nephrons formed. This is compatible with the hypothesis that surface tension is important.

## Discussion

In this report, we have described an improved technique for organ culture of mouse metanephric organ rudiments that is very economical of medium, shows quantitatively better development and also shows cortico-medullary zonation absent in the conventional method. It also uses a transparent substrate, useful for live imaging, that can also be coated easily with experimental custom substrates.

Conventional culture and the low-volume method described here both have the kidney supported at the air-medium interface, with only a thin film of medium covering it; larger volumes of medium, even in exactly the same system, support development significantly less well. There are two obvious *a priori* hypotheses for the importance of the surface: access to oxygen, or the physical compression effect of surface tension. Previous reports of normal development of cultured kidneys in just 3–5% oxygen [47]; [49] make the first of these unlikely. We have shown that lowering the surface tension of the medium using a surfactant results in quantitatively poorer development. This supports the surface tension hypothesis but does not prove it, for the lipid bilayer formed at the surface by the surfactant may also affect the diffusion of gases. To formally prove the biophysical hypothesis that kidneys do better if gently squashed, it would be necessary to vary physical forces only with no effect on chemistry [50]. A possible, though technically difficult, way to achieve this might be to culture kidney rudiments on glass, under large volumes of medium, in a centrifuge.

As well as producing quantitatively better development, the culture system showed a quantitatively strongly reduced expression of markers of cellular stress. As well as arguing for the superiority of the new culture system, this finding highlights a potential but rarely measured problem in organ culture systems; the cells involved might actually be under considerable stress. Some of the molecules they produce as a result of this (and that we measured), such as IFN-γ, will probably only be bioactive in the context of an animal with an immune system but others, such as MMP9 (matrix metalloproteinase-9) and its antagonist Timp-1 (tissue inhibitor of metalloproteinase 1), are important in matrix turnover during development [51] and may therefore lead to culture results not reflecting those obtained *in vivo*. In the kidney itself, there are instances of this: endogenously-produced HGF is needed for collecting duct branching in culture, for example, but HGF^−/−^ mice have normal kidneys [52]. It may be that one explanation of why in vivo and in vitro results do not always agree is a reflection of cellular stress, with measureable induction of protein expression, rather than the often-assumed explanation that the intact body provides some diffusible factor from elsewhere, that can perform the same function as the molecule under study and therefore creates redundancy in vivo, but not in vitro.

In summary, we have presented a culture method that extends the range of questions that can be addressed in culture to include those connected to corticomedullary zonation and loop of Henle formation, and have made culture conditions more economical of medium supplements. As well as making developmental processes more easily visible, this method has the potential to significantly reduce animal use by allowing the control of these aspects of kidney development to be studied in vitro, for example by using siRNAs, rather than by extensive breeding of genetically-modified mice.
